# Supplementary material for: Calypogeia (Calypogeiaceae, Marchantiophyta) in Pacific Asia: Updates from Molecular Revision with Particular Attention to the Genus in North Indochina
Source: Plants (Basel). 2022 Apr 4;11(7):983. doi: 10.3390/plants11070983 (PMC9002642; doi:10.3390/plants11070983)
Supplement: Supplementary file 1 [file plants-11-00983-s001.zip › Figures S1-S3_Condensed trees.pdf]

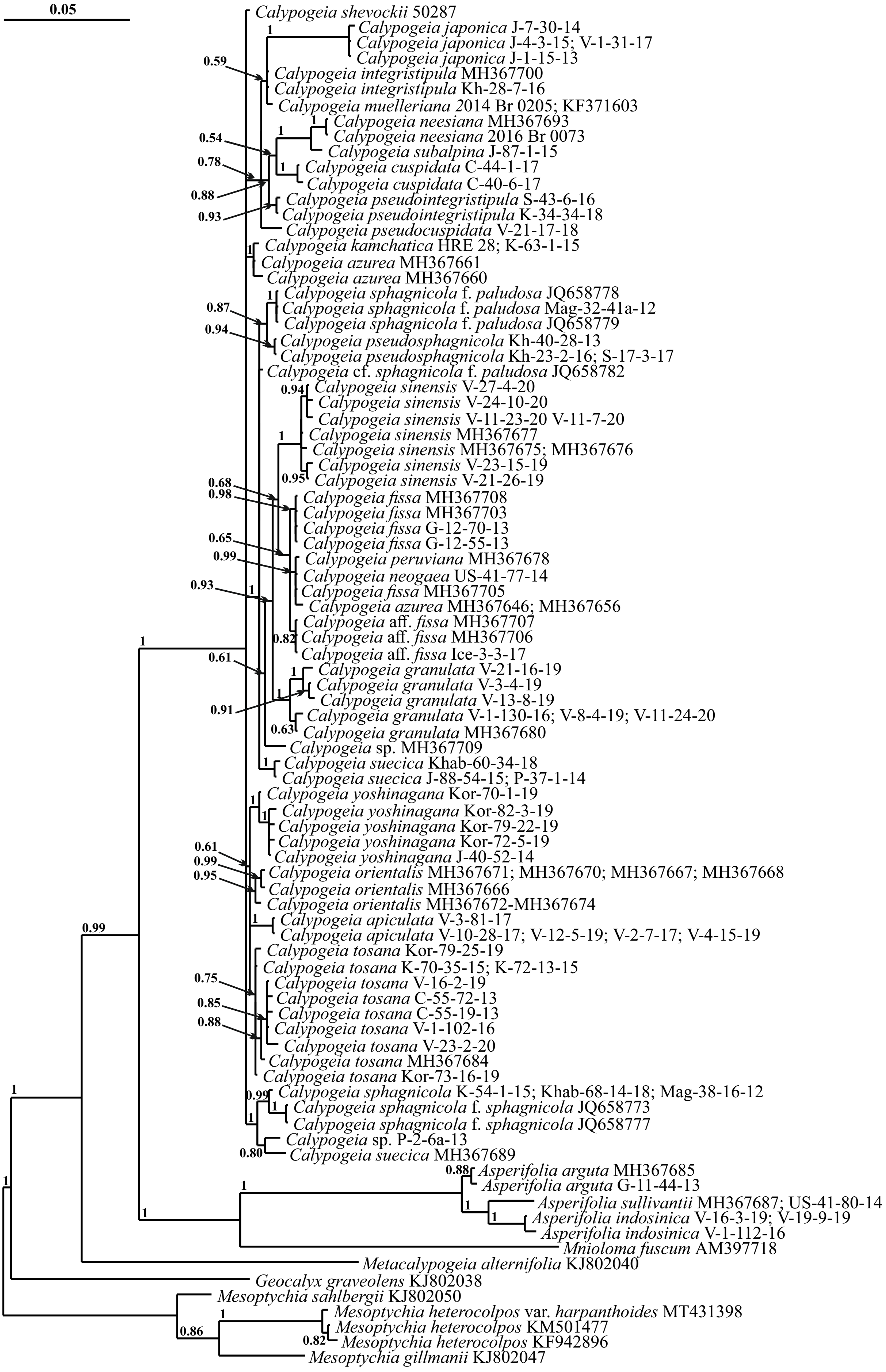

Figure S1. Condensed tree obtained in a Bayesian analysis for the genus *Calypogeia* and related taxa based on *trn*G-intron. The values of Bayesian posterior probabilities more than 0.50 are indicated. Scale bar corresponds to a number of nucleotide substitutions per site.

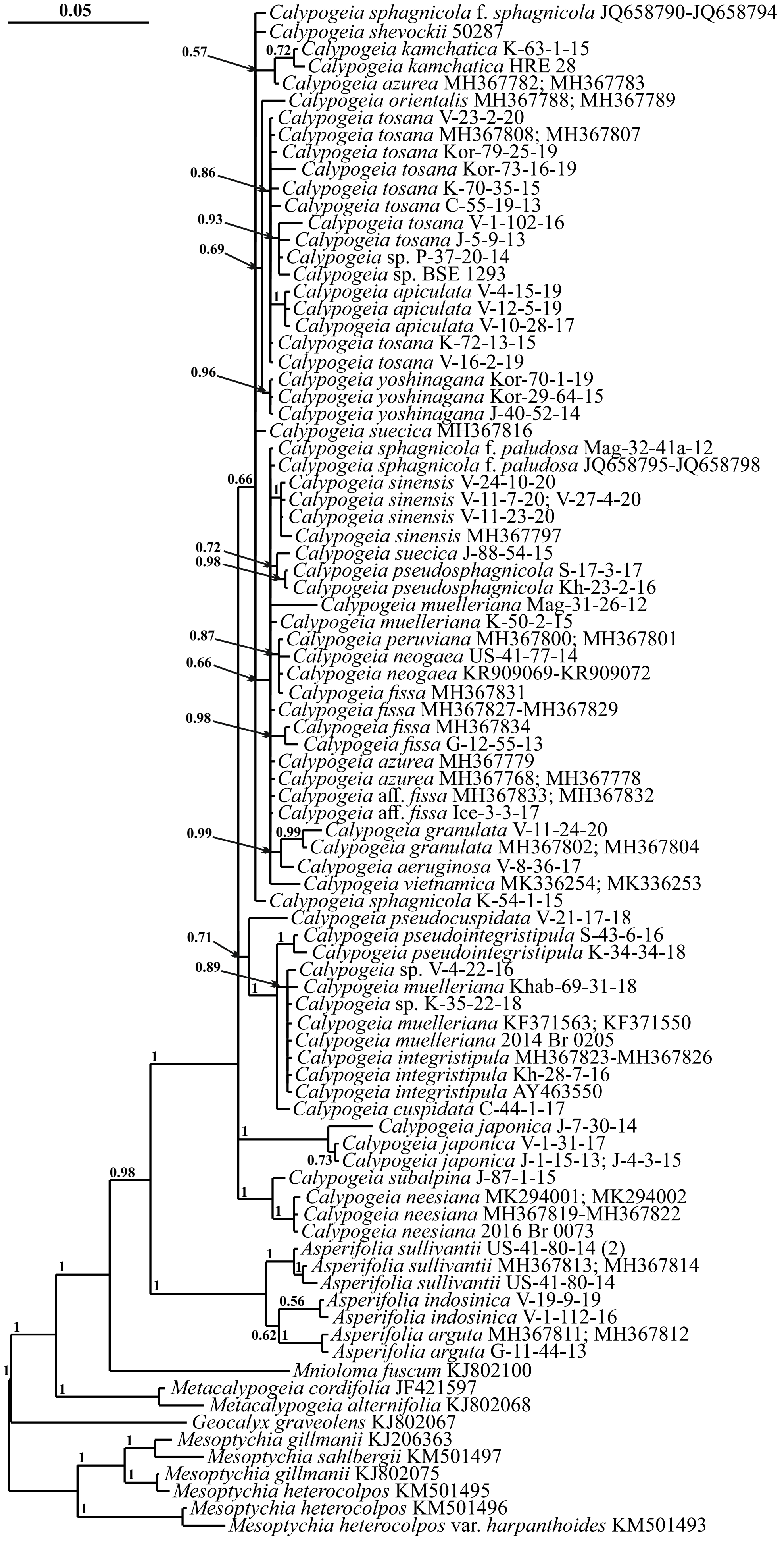

Figure S2. Condensed tree obtained in a Bayesian analysis for the genus *Calypogeia* and related taxa based on *trn*L–F. The values of Bayesian posterior probabilities more than 0.50 are indicated. Scale bar corresponds to a number of nucleotide substitutions per site.

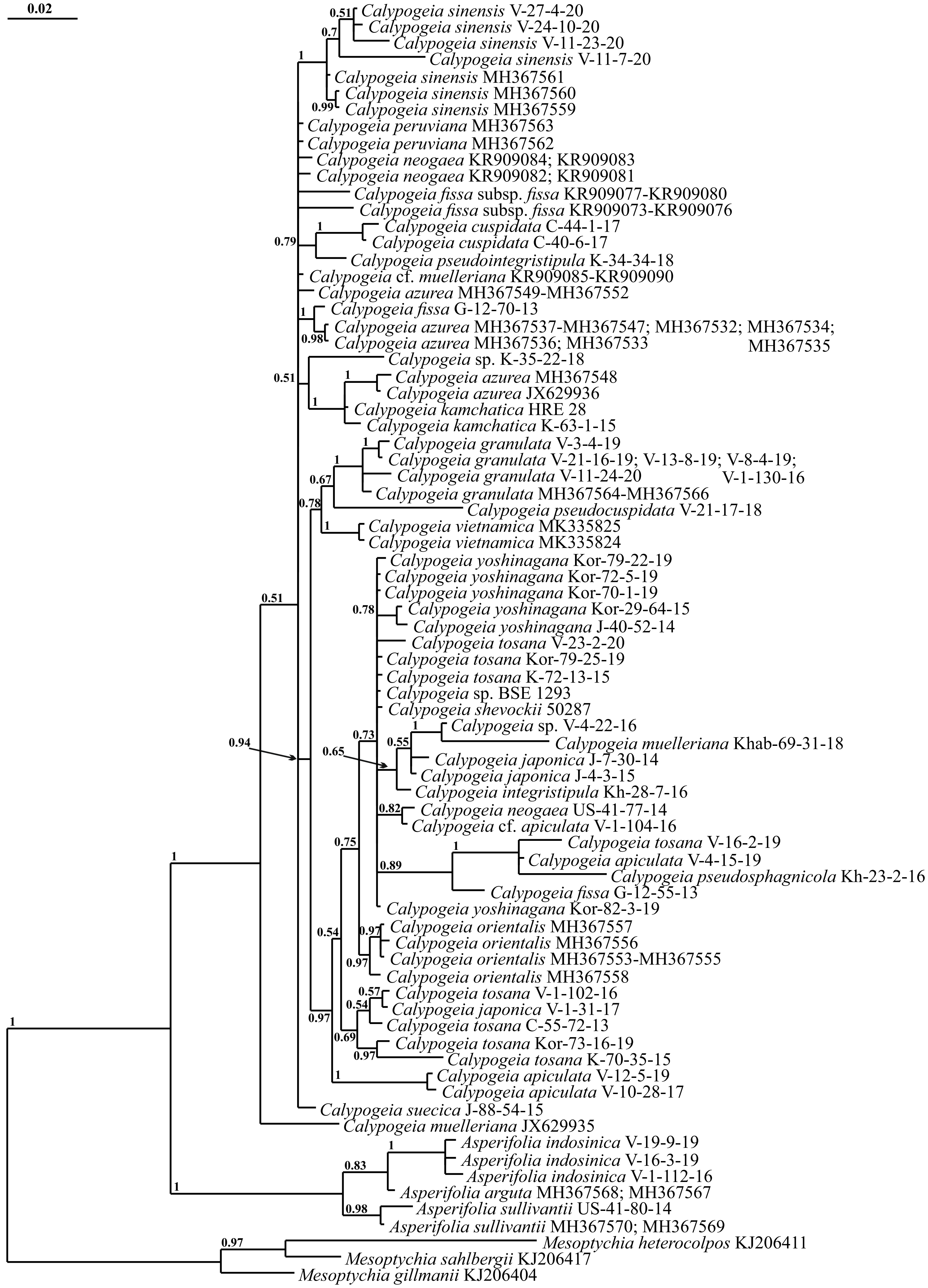

Figure S3. Condensed tree obtained in a Bayesian analysis for the genus *Calypogeia* and related taxa based on ITS 2. The values of Bayesian posterior probabilities more than 0.50 are indicated. Scale bar corresponds to a number of nucleotide substitutions per site.
